# Anti-miR-141-3p maintains homeostasis between autophagy and apoptosis by targeting Yy1 in the fetal lumbosacral defecation center of rats

**DOI:** 10.1016/j.omtn.2024.102163

**Published:** 2024-03-06

**Authors:** Yue Li, Peiqi Liu, Yifan Yao, Weilin Wang, Huimin Jia, Yuzuo Bai, Zhengwei Yuan, Zhonghua Yang

**Affiliations:** 1 Department of Pediatric Surgery, Shengjing Hospital of China Medical University, Shenyang 110004, Liaoning, China; 2 Key Laboratory of Health Ministry for Congenital Malformation, Shengjing Hospital of China Medical University, Shenyang 110004, Liaoning, China

**Keywords:** MT: Oligonucleotides: Therapies and Applications, anorectal malformations, miR-141-3p, Yy1, Adcy3, intraamniotic injection

## Abstract

Anorectal malformations (ARMs) are congenital diseases that lead to postoperative fecal incontinence, constipation, and soiling, despite improvements in surgery; however, their pathological mechanisms remain unclear. Here, we report the role of microRNA-141-3p in maintaining homeostasis between apoptosis and autophagy in the lumbosacral defecation center of fetal rats with ARMs. Elevated microRNA-141-3p expression inhibited YIN-YANG-1 expression by binding its 3′ UTR, and repressed autophagy and triggered apoptosis simultaneously. Then, adenylate cyclase 3 was screened to be the downstream target gene of YIN-YANG-1 by chromatin immunoprecipitation sequencing experiments, and Yin Yang 1 could positively activate the transcription of adenylate cyclase 3 by directly interacting with the motif GAGATGG and ATGG in its promoter. Intraamniotic microinjection of adeno-rno-microRNA-141-3p-sponge-GFP in fetal rats with ARMs on embryonic day 15 restored apoptosis-autophagy homeostasis. These findings reveal that microRNA-141-3p upregulation impaired homeostasis between apoptosis and autophagy by inhibiting the YIN-YANG-1/adenylate cyclase 3 axis, and that intraamniotic injection of anti-microRNA-141-3p helped maintain homeostasis in the lumbosacral defecation center of ARMs during embryogenesis.

## Graphical abstract

**Figure:**
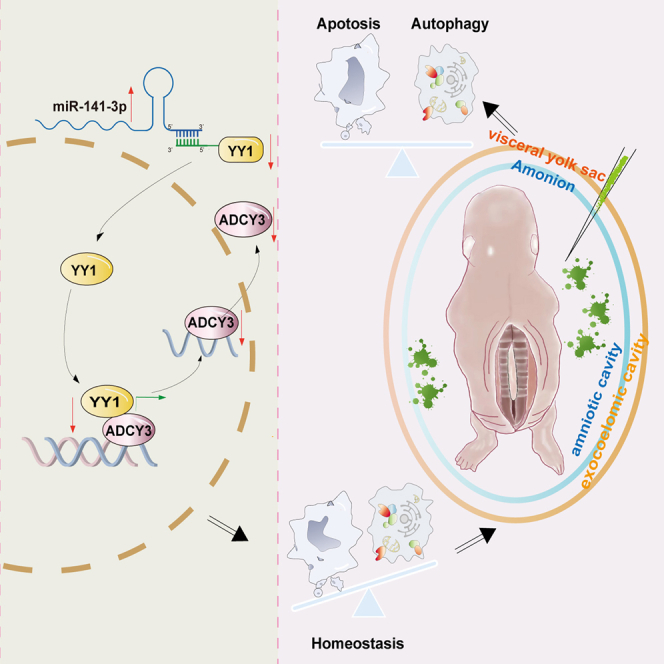

## Teaser

Anorectal malformations are congenital diseases leading to postoperative defecation disorders, despite improvements in surgery. This study revealed that the novel microRNA-141-3p/Yin Yang 1/adenylate cyclase 3 axis dysregulated apoptosis and autophagy homeostasis, and intraamniotic injection of anti-microRNA-141-3p helped maintain homeostasis in the defecation center of rats with anorectal malformations during embryogenesis.

## Introduction

Anorectal malformations (ARMs) occur in 1 in 3,000–5,000 newborns and comprise various defects ranging from minor to complex cloacal malformations.^1^^,^^2^ Therefore, although cloacal anomalies can be addressed by appropriate surgical repair, lifelong management of continence is needed because abnormal lumbosacral spinal cord function causes outcomes that cannot be predicted based on the type of ARMs involved. Therefore, researching the lumbosacral medulla, where the defecation center resides, is crucial for predicting and improving postoperative defecation function in patients with ARMs.

Many pathologic findings have been obtained by studying the lumbosacral defecation centers in ARMs. Previously, we identified defective motoneurons, sacral parasympathetic nuclei, and sensory neurons innervating the sphincter and anorectum in the defecation center.^3^^,^^4^^,^^5^ Furthermore, we found that neurons decreased due to extensive apoptosis in rat embryos with ARMs.^6^ Therefore, extensive apoptosis, leading to fewer neurons in the defecation control center, likely contributes to defecation dysfunction after the surgery.

Apoptosis and autophagy help maintain cellular homeostasis.^7^^,^^8^ However, the relationship between autophagy and apoptosis in ARMs is unknown. Previously, we showed that abnormal noncoding RNA expression promoted maldevelopment of the lumbosacral defecation center in ARMs based on comparative transcriptome analysis of lumbosacral spinal cord tissues from rat embryos with ethylene thiourea (ETU)-induced ARMs and those from normal embryos.^9^ We also identified microRNA (miR)-200 family members that drove lumbosacral spinal cord dysplasia in fetal rats with ARMs through a microRNA transcription factor-mRNA network. miR-141-3p, which is an important member of the miR-200 family, is expressed during fetal development and displays multiple biological functions. miR-141-3p upregulation in decidual natural killer cells could be associated with unexplained recurrent spontaneous abortions,^10^ and an miR-141-3p inhibitor decreased apoptosis and repressed activation of the mitochondrial apoptosis pathway in palmitic acid-treated H9c2 cells.^11^ miR-141-3p overexpression significantly suppressed ectopic endometrial stromal cell proliferation, while promoting apoptosis.^12^ Conversely, it is also reported that miR-141-3p inhibition induced apoptosis, suppressed proliferation, and reduced profibrotic markers in hepatic stellate cells by blocking the PTEN-AKT pathway.^13^

In addition, miR-141-3p serves dual functions in autophagy. Silencing miR-141-3p in four esophageal cancer cell lines enhanced autophagy and decreased the oncogenic capacity.^14^ miR-141-3p overexpression aggravated fibrosis and restrained autophagy in human mesangial cells incubated with high glucose.^15^ In contrast, autophagy induced by miR-141-3p in placental trophoblasts may be associated with preeclampsia.^16^ The homeostasis between apoptosis and autophagy jointly determines the fates of cells. However, the mechanism whereby miR-141-3p affects homeostasis in lumbosacral spinal cord dysplasia and the functional role of intraamniotic anti-miR-141-3p injection in apoptosis and autophagy in ARMs during embryogenesis remain unclear.

The aim of this study was to investigate the function of miR-141-3p and to study the possible functional role of anti-miR-141-3p in ARMs. These findings provide insights into the mechanism of defecation center development in ARMs and lay the foundation for potential therapeutic targets to improve postoperative defecation function.

## Results

### miR-141-3p and YIN-YANG-1 (YY1) expression were dysregulated in the lumbosacral spinal cords of fetal rats with ARMs on embryonic day (E) 17

**Figure 1:**
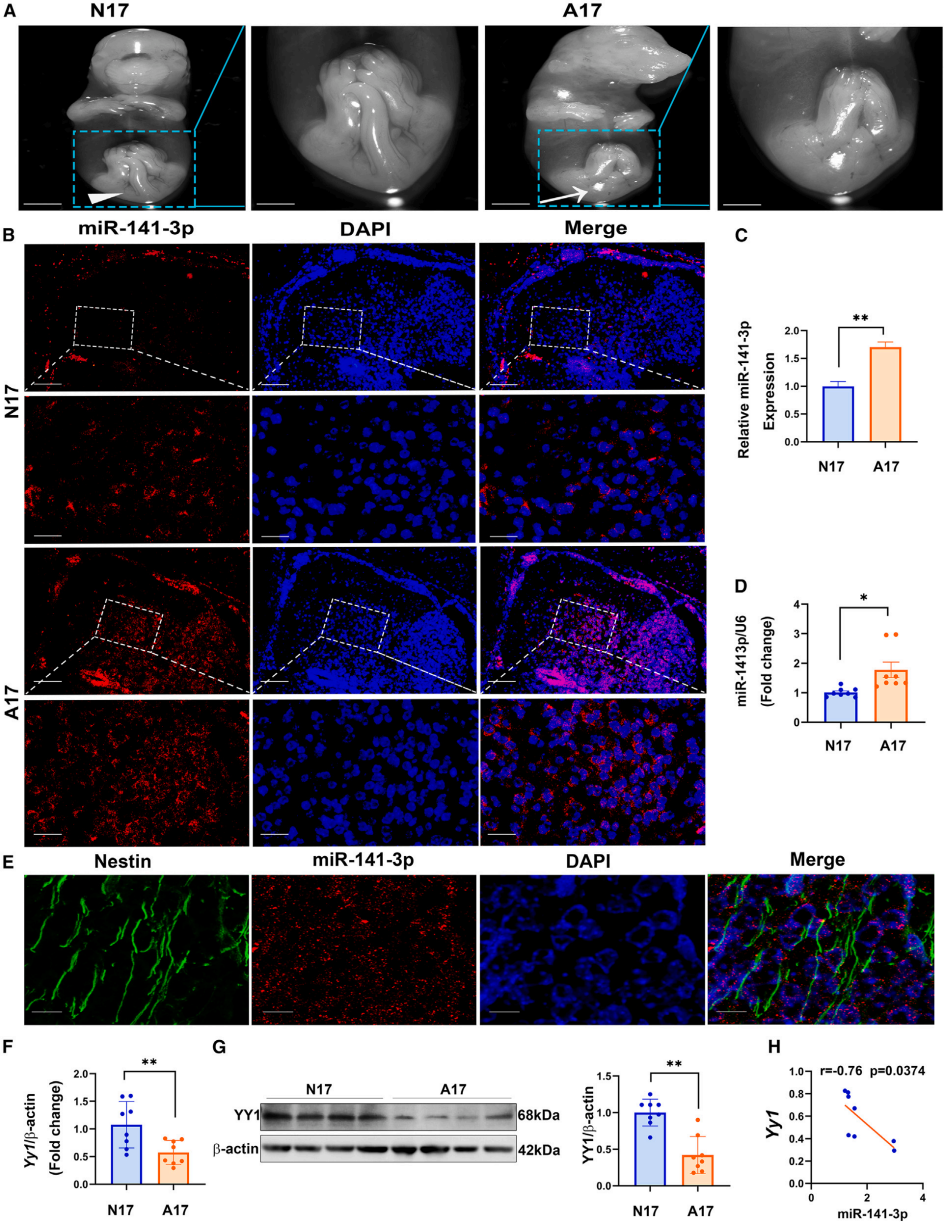
miR-141-3p and YY1 expression in a fetal rat model of ETU-induced ARMs (A) Gross morphology of fetal rats in the control and ARMs groups, observed under a stereomicroscope on E17 (arrow: no anus and tail in ARMs fetal rat; arrowhead: normal anus with tail; scale bar, 2 mm). (B) The expression patterns of miR-141-3p (red) detected by FISH assays in lumbosacral spinal cords of control and ARMs embryos, combined with nuclei staining using DAPI (blue) at 10× (scale bar, 50 μm) and 40× (scale bar, 12.5 μm) (oil). (C) Quantification of miR-141-3p expression by AOD analysis in lumbosacral spinal cords of control and ARMs embryos. (D) Relative miR-141-3p-expression levels in the lumbosacral spinal cords of normal rat fetuses and rat fetuses with ETU-induced ARMs on E17 by qRT-PCR (n = 8). (E) Colocalization of miR-141-3p (red) and NESTIN (green) in the spinal cord of fetal rat detected by combined FISH/immunostaining at 80× (scale bar, 6.25 μm). (F) Relative expression levels of *Yy1* mRNA in the lumbosacral spinal cords of normal rat fetuses and rat fetuses with ETU-induced ARMs on E17 (n = 8). (G) Comparison of the protein-expression levels of YY1 in lumbosacral spinal cords from rat fetuses on the control and ARMs groups on E17 (n = 8). (H) Correlation analysis for miR-141-3p and *Yy1* RNA-expression levels in rat fetuses with ARMs on E17. The data shown represent the results of 3 experiments. The data shown indicate the mean values of triplicate experiments ± SD. (N17, normal fetal rats on E17; A17: fetal rats with ARMs on E17.) ∗p < 0.05, ∗∗p < 0.01, relative to the control group.

Fetal rat embryos with ARMs (collected from ETU-treated dams) had an unobservable anus without other visible abnormalities under a dissection microscope on E17, whereas an observable anus was found in control rat embryos (Figure 1A). Our previous transcriptome analysis of the spinal cord expression profiles of fetuses with ARMs revealed miR-141-3p upregulation and downregulation of the target gene encoding transcription factor *Yy1*, with a highly negative correlation score (−0.953) via bioinformatics analysis (Miranda and TargetScan), which probably contributed to lumbosacral spinal cord dysplasia in fetal ARMs.^9^ Therefore, the expression of miR-141-3p was analyzed by fluorescence *in situ* hybridization (FISH) assay and qRT-PCR analyses. The FISH assay in the lumbosacral spinal cords of rat embryos further showed that miR-141-3p was predominantly localized in the cytoplasm. A higher intensity of miR-141-3p expression is seen and quantified in ARMs fetus (Figures 1B and 1C). The results of qRT-PCR showed that miR-141-3p was consistently expressed at higher levels in fetal rats with ETU-induced ARMs on E17 than in control embryos (Figure 1D). To further explore whether miR-141-3p expressed in neurons, we performed combined FISH/immunostaining. miR-141-3p was found in NESTIN^+^ cells (Figure 1E), indicating the possible role of miR-141-3p in neural stem and neural progenitor cells during embryonic development. Meanwhile, miR-141-3p detected in NESTIN^−^ cells probably played a part in the neural cells through paracrine. Notably, miR-141-3p were also observed in their surrounding cells without NESTIN expression, implying that miR-141-3p can also affect NESTIN^+^ cells by altering the microenvironment. The mRNA and protein expression levels of YY1 were lower in lumbosacral spinal cord tissues of the ARMs group on E17 than in those of the control group (Figures 1F and 1G). Then, we performed Pearson correlation analysis on the mRNA expression levels of miR-141-3p and *Yy1* on E17, and the results showed a significantly strong negative correlation between them (*R* = −0.736, p = 0.0374; Figure 1H), which was consistent with the above-mentioned predicted bioinformatics results. These results indicate that *Yy1* is a potential target gene of miR-141-3p.

### miR-141-3p interacted with 3ʹ UTR of *Yy1* mRNA and inhibited its expression

**Figure 2:**
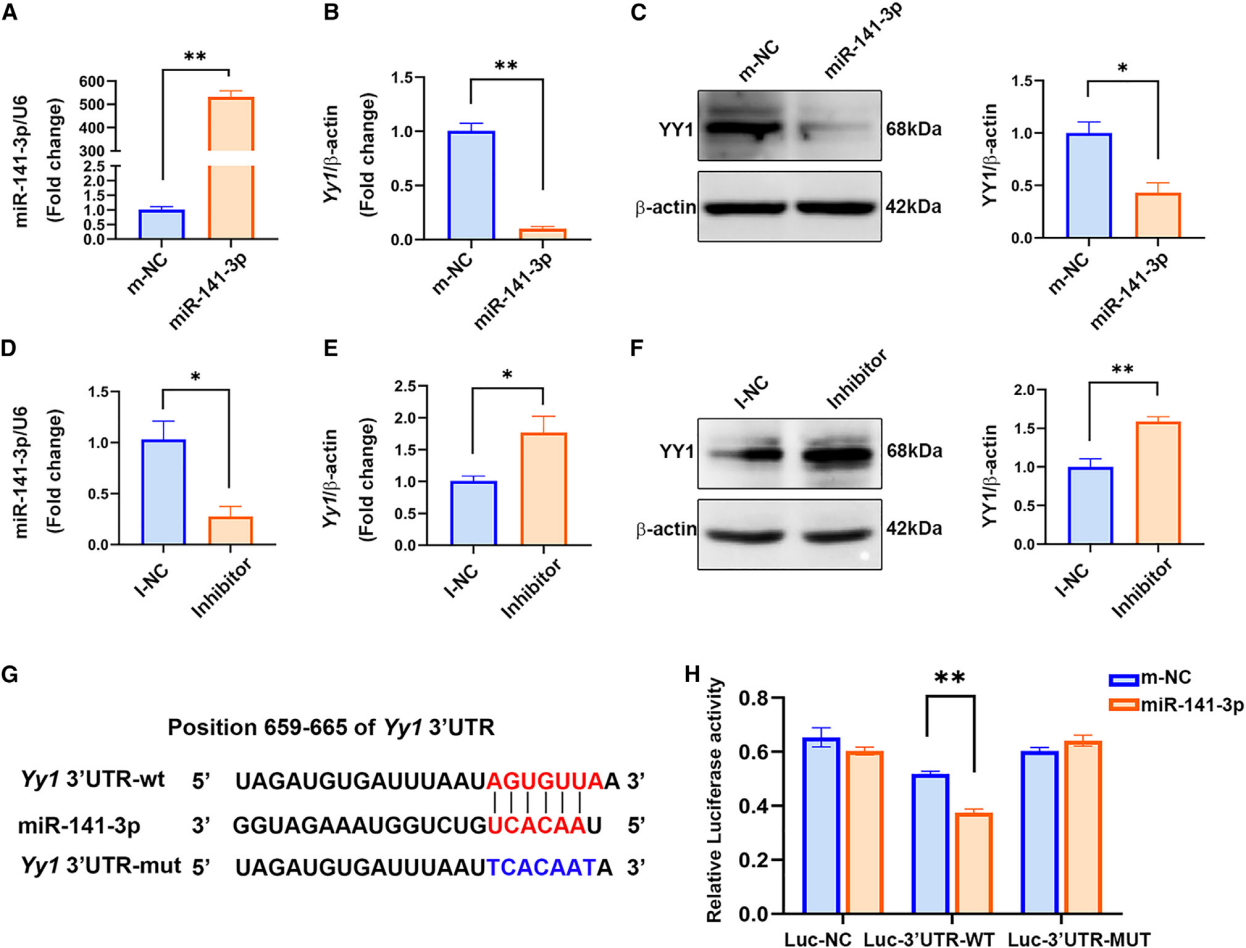
miR-141-3p suppressed the expression of YY1 by binding to its 3ʹ UTR (A and B) mRNA levels of miR-141-3p (A) and *Yy1* (B) in C17.2 cells transfected with an miR-141-3p mimic or a control mimic for 48 h. (C) Protein-expression levels of YY1 in C17.2 cells transfected with 50 nmol miR-141-3p mimic or the control mimic for 48 h. (D and E) Changes in miR-141-3p (D) and *Yy1* (E) mRNA expression levels after transfection with 50 nmol miR-141-3p inhibitor or the control inhibitor for 48 h. (F) YY1 protein expression in C17.2 cells was detected by western blotting after the cells were transfected with 50 nmol miR-141-3p inhibitor. (G) WT mouse *Yy1* mRNA and mutations in potential target sites for miR-141-3p in the 3′ UTR of mouse *Yy1* mRNA, as predicted by TargetScan. (H) Relative luciferase activity levels of the WT-Luc and Mut-Luc reporters after miR-141-3p transfection. The data shown indicate the mean values of triplicate experiments ± SD. ∗p < 0.05, ∗∗p < 0.01, relative to the control group.

To validate *Yy1* as a downstream target of miR-141-3p, we used the miRNA target-prediction programs TargetScan and miRDB and confirmed that miR-141-3p may directly target the 3′ UTR region of *Yy1*. After transfecting an miR-141-3p mimic into C17.2 cells (Figure 2A), qRT-PCR and western blot analyses revealed that the mRNA and protein expression levels of YY1 decreased significantly (Figures 2B and 2C). Conversely, after transfecting an miR-141-3p inhibitor into C17.2 cells (Figure 2D), the mRNA and protein expression levels of YY1 increased significantly (Figures 2E and 2F). Next, we validated the interaction between miR-141-3p and *Yy1* by performing luciferase reporter assays. Analysis with the TargetScan database showed that the *Yy1* 3′ UTR has a putative binding site for miR-141-3p (Figure 2G). We performed assays with a wild-type (Luc-3′-UTR-WT) or mutated *Yy1* 3′ UTR-coupled luciferase reporter (Luc-3′ UTR-MUT). miR-141-3p clearly inhibited the luciferase activity of *Yy1* Luc-3′ UTR-WT in C17.2 cells, whereas no significant change was observed in the mutant *Yy1* 3′ UTR, indicating that *Yy1* is a target of miR-141-3p (Figure 2H). Collectively, these results indicate that miR-141-3p directly bound to *Yy1* and downregulated its expression.

### Genome-wide YY1 binding was mapped by chromatin immunoprecipitation sequencing (ChIP-seq) and *Adcy3* was selected as a candidate downstream gene of YY1

**Figure 3:**
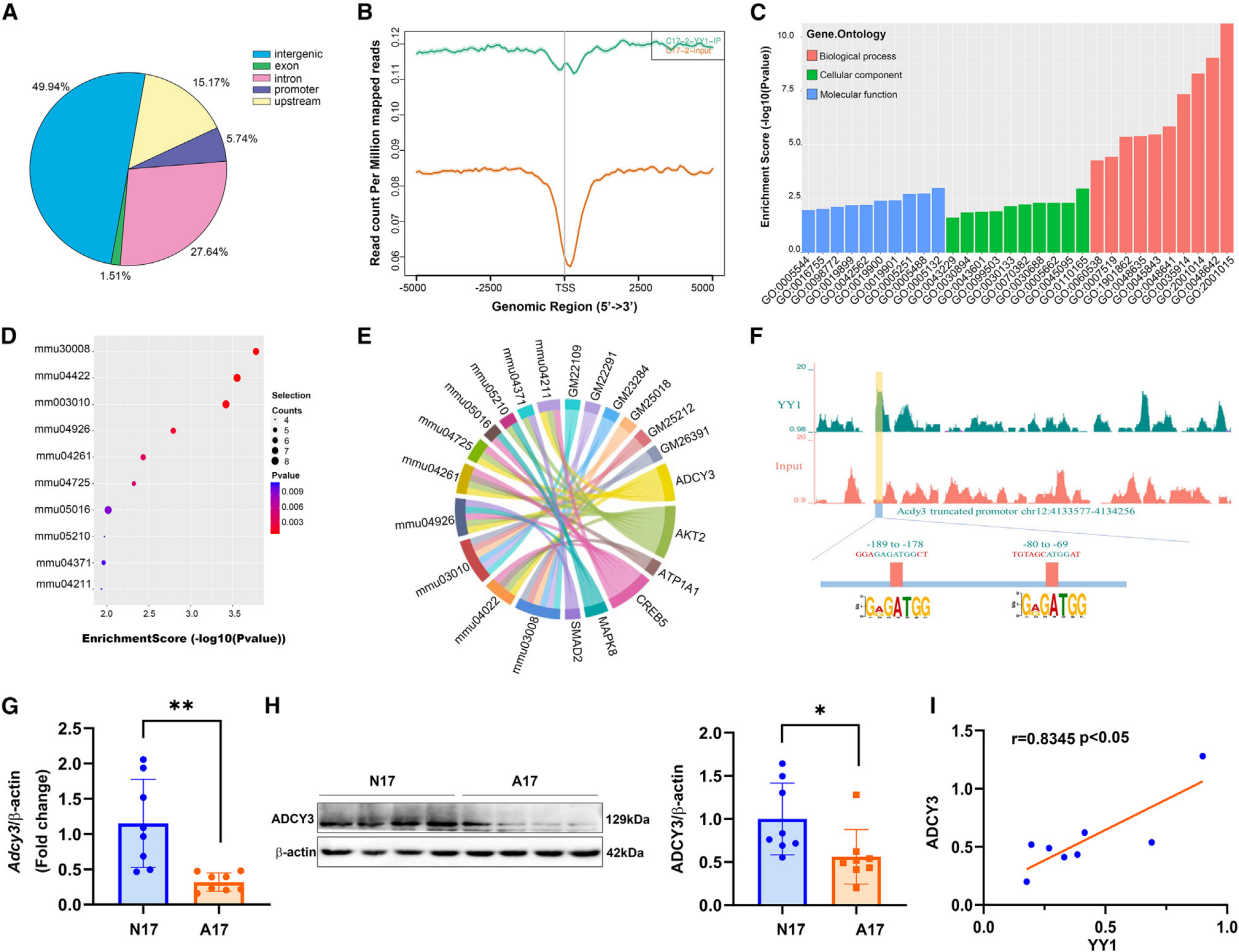
Genome-wide YY1 occupancy in C17.2 cells and decreased ADCY3 expression in ARMs embryos (A) Distribution of YY1-binding peaks in C17.2 cells, located within ±2 kb of the TSS. (B) Frequency of peak regions for YY1-binding sites located ±5 kb from the TSS in C17.2 cells. (C) GO analysis of genes whose promoter regions bound YY1 (detailed GO terms provided in Table S1). (D) The top 10 enriched KEGG pathway terms associated with genes whose promoter regions bound YY1 in C17.2 cells. The pathway terms are shown on the y axis and the enrichment scores for the top 10 enriched terms are shown on the x axis. (E) Chord diagram showing the top 10 most enriched KEGG pathway terms associated with genes whose promoter regions bound YY1 (detailed KEGG terms provided in Table S2). (F) View of YY1 ChIP-seq density profiles for the *Adcy3* promoter region and *de novo*-motif predictions of DNA sequences containing an enrichment for YY1-binding regions within ±1 kb of the *Adcy3* TSS. (G) mRNA levels of *Adcy3* in the lumbosacral spinal cords of normal rat fetuses and rat fetuses with ETU-induced ARMs at E17 (n = 8). (H) Abundance of ADCY3 protein expression in the lumbosacral spinal cords of rat fetuses with ARMs on E17 (n = 8). (I) Protein-expression correlation analysis for YY1 and ADCY3 in rat fetuses with ARMs on E17. The data shown represent the mean values of triplicate experiments ± SD. ∗p < 0.05, ∗∗p < 0.01, relative to the control group.

YY1 is an important transcription factor in the nervous system.^17^ To gain global insights into the role of YY1 in neurogenesis in ARMs, we performed unbiased, genome-wide studies of YY1-binding sites in C17.2 cells by ChIP and subsequent deep sequencing using an anti-YY1 antibody. Precipitated DNA fragments, ranging from 200 to 1,500 nt in length, were subjected to high-throughput sequencing. We identified 393 YY1-binding sites with high confidence that were distributed primarily around the promoter regions (±2 kb from the transcription start sites [TSSs]) of the corresponding genes (159 coding genes and noncoding genes) (Figure 3A). The promoter-binding peaks were highly enriched in regions 1 kb upstream and downstream of the TSSs (Figure 3B).

To gain further insights into the functional relevance of YY1 binding, promoter peak-related genes were subjected to Gene Ontology (GO) analysis. The target genes were significantly enriched for GO terms such as type I interferon receptor binding, cellular anatomical entities, keratin filaments, negative regulation of skeletal muscle cell differentiation, and negative regulation of skeletal muscle tissue development, indicating that YY1 may serve a key role in development (Figure 3C; Table S1). Pathway analysis of the promoter peaks related to YY1 binding was performed using the Kyoto Encyclopedia of Genes and Genomes (KEGG) database. The top 10 enriched pathways are shown in Figure 3D, and detailed terms of KEGG are provided in Table S2. Among these 10 pathways, adenylate cyclase 3 (*Adcy3*) was associated with enrichment for six pathways (Figure 3E), indicating its important biological role in mediating YY1 function. The location of the YY1-binding peak in the promoter region of *Adcy3* was identified as being ∼576 bp upstream to 104 bp downstream of the TSS. Moreover, two binding-motif sites were identified between nucleotide positions −189 to −178 and −80 to −69 with sequences of 5′-GAGATGG-3′ and 5′-ATGG-3′ (Figure 3F). Subsequently, we explored ADCY3 expression in the lumbosacral spinal cords of rat embryos with ARMs via qRT-PCR and western blot analyses, finding that the mRNA and protein expression levels of ADCY3 were markedly lower on E17 (Figures 3G and 3H). Moreover, ADCY3 expression showed a strong positive correlation with YY1 protein expression in rat embryos with ARMs on E17 (*R* = 0.8345, p < 0.05; Figure 3I).

### YY1 transcriptionally activated ADCY3 in C17.2 cells

**Figure 4:**
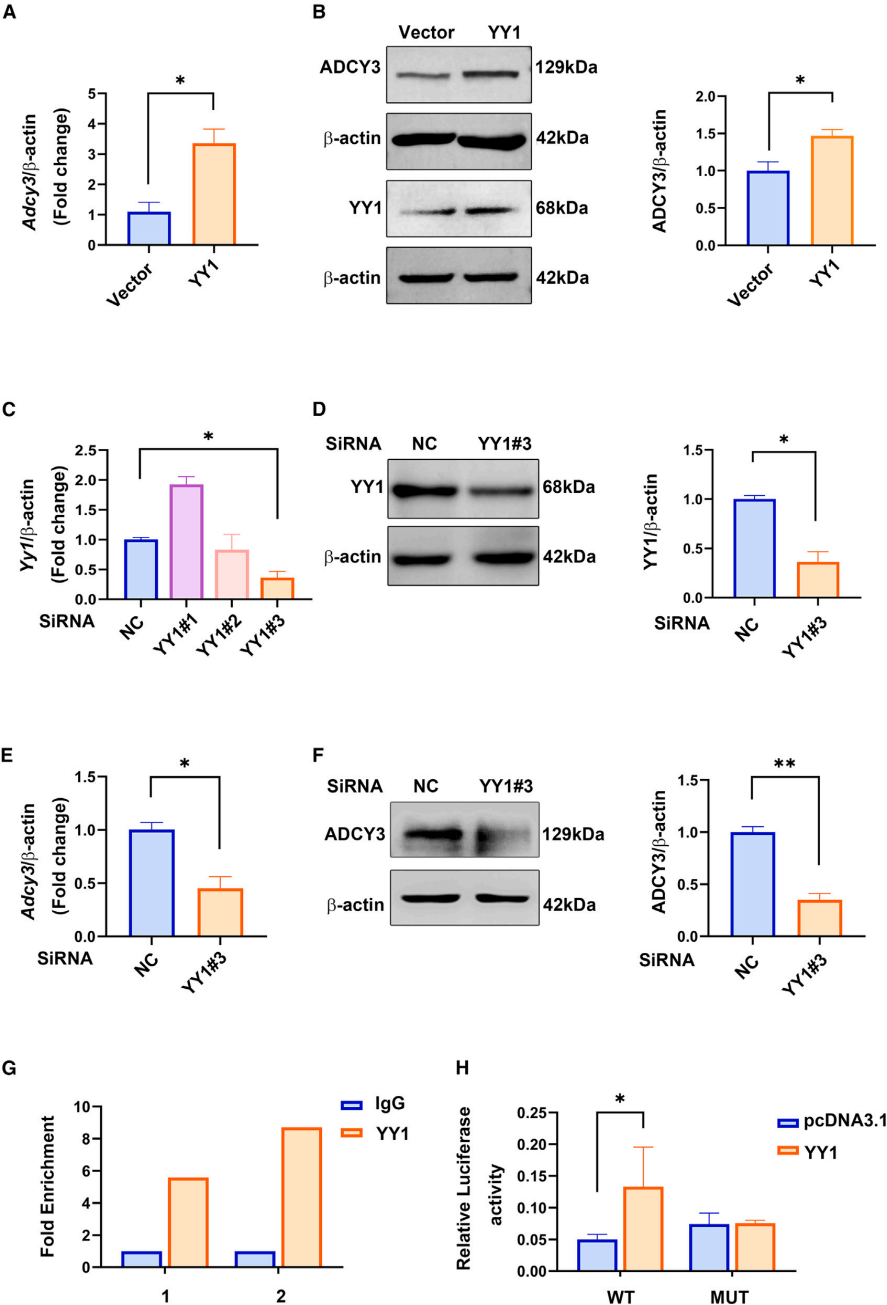
YY1 positively regulated ADCY3 transcription in C17.2 cells (A and B) The mRNA (A) and protein (B) levels of ADCY3 in C17.2 cells after transfecting them with the YY1 expression plasmid or the control vector. (C and D) Levels of YY1 mRNA (C) and protein (D) after transfecting C17.2 cells with YY1 siRNA or control siRNA. (E and F) The protein (E) and mRNA (F) levels of ADCY3 in C17.2 cells after RNAi against YY1. (G) ChIP-qPCR verification of YY1 binding to motifs 1 and 2 in the *Adcy3* promoter region (see Figure 3F) using chromatin isolated from C17.2 cells. (H) Luciferase activities were measured after cotransfecting C17.2 cells with a vector encoding the WT or MUT *Adcy3* promoter, with or without the YY1 expression plasmid. The data shown represent the mean values of triplicate experiments ± SD. ∗p < 0.05, ∗∗p < 0.01, relative to the control group.

Next, we investigated the modulation of YY1 on ADCY3 expression. The mRNA and protein levels of YY1 in C17.2 cells increased after they were transfected with a YY1 overexpression plasmid (Figure S1). Moreover, ADCY3 mRNA and protein expression increased significantly following YY1 overexpression (Figures 4A and 4B). Furthermore, three small interfering RNAs (siRNAs) against YY1 were used to silence *Yy1* mRNA expression, and the results have shown that siYY1 #3 could silence the mRNA level of *Yy1* significantly and was selected for further experiments (Figure 4C). Similar to that in the mRNA expression data, siYY1 #3 decreased YY1 protein expression (Figure 4D). Altogether, siYY1 #3 was found to be more effective than siYY1 #1 and siYY1 #2 at silencing YY1 expression. Similarly, the mRNA and protein expression levels of ADCY3 were downregulated in C17.2 cells following YY1 silencing with siYY1 #3 (Figures 4E and 4F). Collectively, these results suggest that ADCY3 is a downstream target of the transcription factor YY1 and that it can be transcriptionally activated by YY1.

To explore whether YY1 can bind to the *Adcy3* promoter and directly activate ADCY3, a ChIP assay was performed with anti-YY1 antibodies and crosslinked chromatin obtained from C17.2 cells. Indeed, qPCR analyses using primers specific for motifs 1 and 2 of the *Adcy3* locus confirmed that YY1 was recruited to the *Adcy3* promoter (Figure 4G). Dual-luciferase reporter assays were used to test the functionality of this putative YY1-binding site by overexpressing YY1 in C17.2 cells together with a luciferase plasmid containing the *Adcy3* promoter. Substantial induction of luciferase activity occurred when both YY1 and the WT *Adcy3* promoter were introduced, but not when the putative YY1-binding site was mutated (Figure 4H). Thus, the ChIP and luciferase assays validated the direct binding of YY1 to the *Adcy3* promoter and confirmed the critical role of YY1 in the transcriptional activation of ADCY3.

### Apoptosis triggered by miR-141-3p was mediated by the YY1-ADCY3 axis

**Figure 5:**
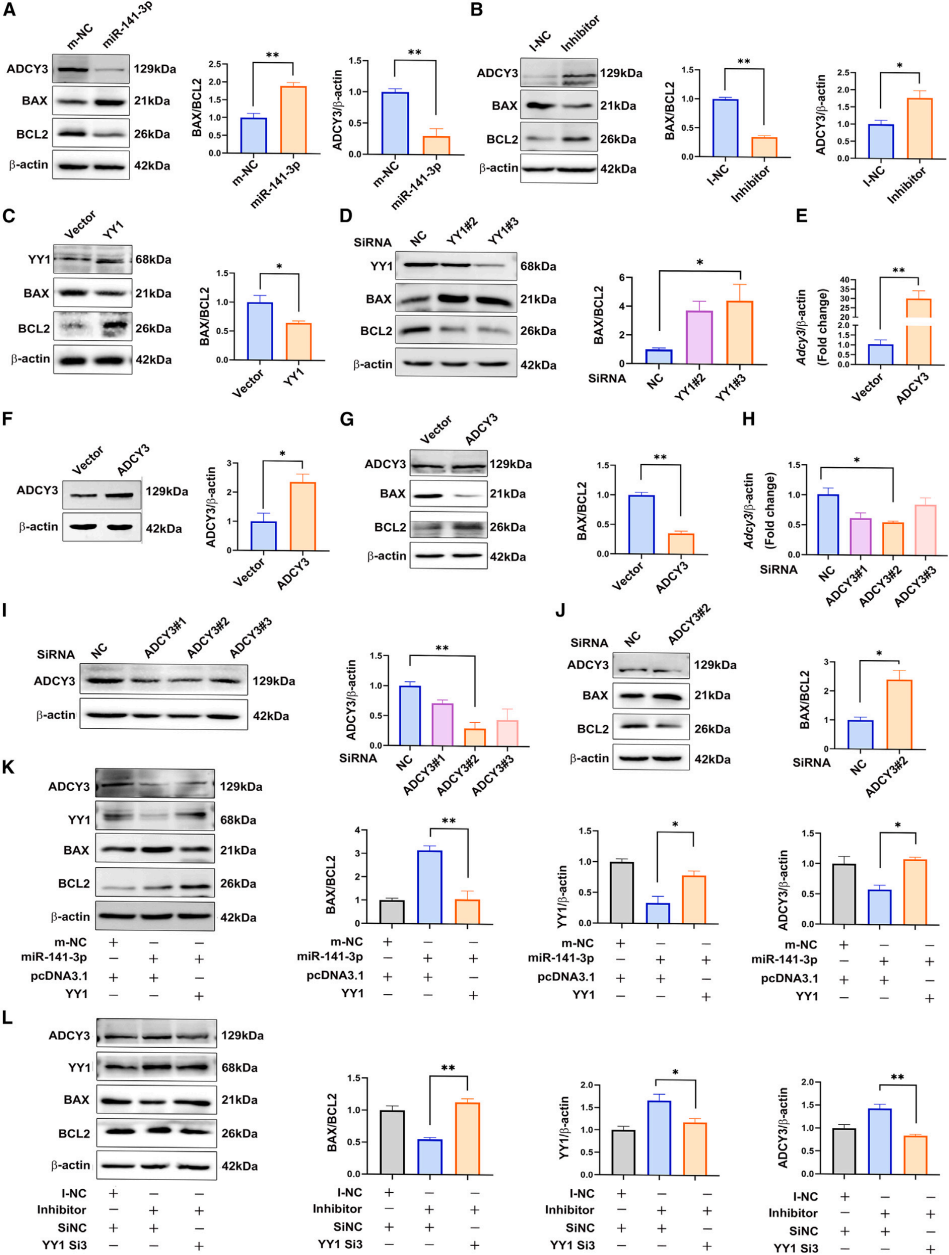
Apoptosis was triggered by miR-141-3p-based inhibition of the YY1-ADCY3 axis (A) Protein levels of apoptosis markers (BAX and BCL-2) and ADCY3 were estimated by western blotting for C17.2 cells transfected with the miR-141-3p mimic or a control mimic. (B) Protein levels of apoptosis markers (BAX and BCL-2) and ADCY3 in cells transfected with an miR-141-3p inhibitor or a control inhibitor in C17.2 neural stem cells. (C) Changes in the BAX:BCL-2 protein expression ratio observed after transfection with the YY1 expression plasmid or the control vector in C17.2 neural stem cells. (D) Changes in the BAX:BCL-2 protein expression ratios detected in cells transfected with YY1 siRNA #2, siRNA #3, or a control siRNA in C17.2 neural stem cells. (E and F) Changes in the mRNA expression (E) and protein expression (F) levels of ADCY3 in C17.2 neural stem cells after transfecting them with the ADCY3 expression plasmid or the control vector. (G) Changes in the BAX and BCL-2 protein expression levels in C17.2 cells transfected with the ADCY3 expression plasmid or the control vector. (H and I) Changes in the mRNA expression (H) and protein expression (I) levels of ADCY3 in C17.2 cells transfected with ADCY3 siRNA or control siRNA. (J) Change in the BAX:BCL-2 protein ratio in cells transfected with ADCY3 siRNA #3 or the control siRNA. (K) Changes in the expression levels of the BAX, BCL-2, and ADCY3 proteins after cotransfection with the miR-141-3p mimic and the YY1 expression plasmid or control vector. (L) Changes in the BAX:BCL-2 protein ratio and ADCY3 protein levels after transfection with an miR-141-3p inhibitor, with or without YY1 siRNA #2. The data shown indicate the mean values of triplicate experiments ± SD. ∗p < 0.05, ∗∗p < 0.01, relative to the control group.

Previously, we showed that excessive apoptosis occurred in the lumbosacral spinal cords of rat fetuses with ARMs^6^; therefore, in this study, we quantified BAX, BCL2, and ADCY3 expression in C17.2 cells after transfecting them with miR-141-3p mimic or inhibitor. The miR-141-3p-mimic transfectants showed increased expression of the proapoptotic protein BAX but inhibited the expression of ADCY3 and the antiapoptotic protein BCL-2 (Figure 5A). In contrast, the miR-141-3p-inhibitor transfectants showed the opposite effects in terms of BAX, BCL2, and ADCY3 (Figure 5B). Next, we investigated whether the abundance of YY1 affected embryogenesis through apoptosis. YY1 overexpression significantly decreased the BAX:BCL-2 ratio, whereas YY1 silencing increased the ratio in C17.2 cells, especially when using siYY1 #3 (Figures 5C and 5D).

To determine the effect of ADCY3 on apoptosis, the BAX:BCL-2 ratio was quantified after downregulating or upregulating ADCY3 in C17.2 cells. The mRNA and protein expression levels of ADCY3 increased significantly after transfection with ADCY3 overexpression plasmid (Figures 5E and 5F), which was accompanied by a decreased BAX:BCL-2 ratio (Figure 5G). The BAX:BCL-2 ratio increased (Figure 5J) when ADCY3 mRNA and protein expression were significantly downregulated by ADCY3 silencing with siADCY3 #2 (Figures 5H and 5I). To further characterize whether miR-141-3p-induced apoptosis and ADCY3 expression differences were mediated by YY1, both YY1 and miR-141-3p mimics were transfected into C17.2 cells. The miR-141-3p mimic induced extensive apoptosis and decreased ADCY3 expression, which were reversed by YY1 overexpression (Figure 5K). Consistently, decreased apoptosis and increased ADCY3 expression induced by the miR-141-3p inhibitor were abrogated by siYY1 #3 (Figure 5H), demonstrating that miR-141-3p-dependent apoptosis was mediated by the YY1-ADCY3 axis. Thus, our data suggest that increased miR-141-3p levels promote apoptosis by inhibiting the YY1-ADCY3 axis, thereby leading to maldevelopment of the lumbosacral spinal cords in rat embryos with ARMs.

### miR-141-3p repressed autophagy by inhibiting the YY1-ADCY3 axis

**Figure 6:**
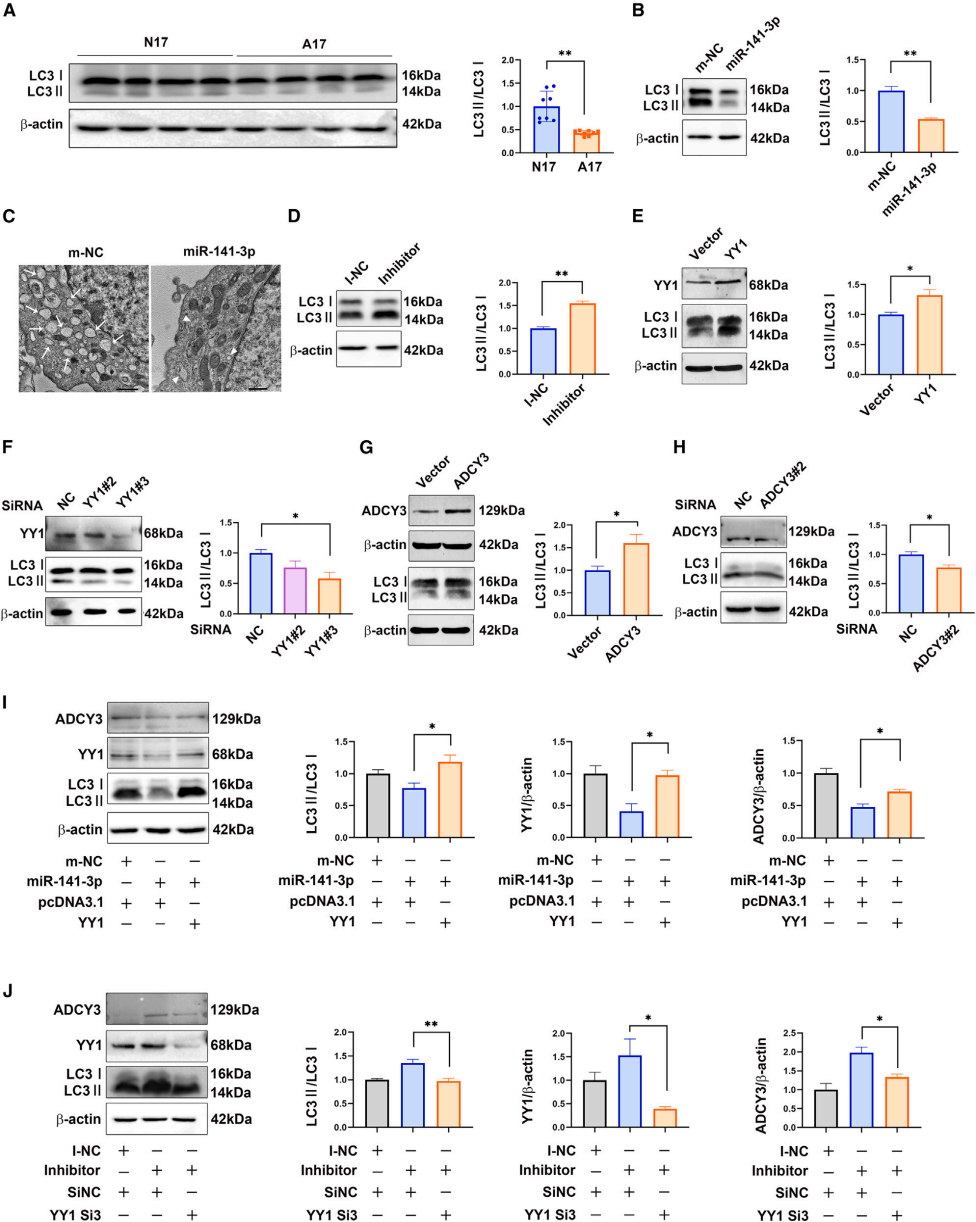
miR-141-3p activated the YY1-ADCY3 axis, which impaired autophagy (A) Conversion of LC3-I to LC3-II in the lumbosacral spinal cords of rat fetuses with ETU-induced ARMs, compared with those in the control group. (B) Protein expression levels of LC3-I and LC3-II in cells transfected with the miR-141-3p mimic or the control mimic were estimated by western blotting. (C) Greater and increased autolysosome formation (white arrow; scale bar, 1 μm) in C17.2 cells transfected with the miR-141-3p mimic than in those transfected with the control mimic (white arrowhead, scale bar, 500 nm), as revealed by TEM analysis. (D) Changes in the LC3-I and LC3-II protein expression levels in cells transfected with the miR-141-3p or control inhibitor. (E) Difference in the conversion of LC3-I to LC3-II induced by transfection with the YY1 expression plasmid versus that observed after transfection with the control vector. (F) Changes in the conversion of LC3-I to LC3-II in C17.2 cells transfected with YY1 siRNA #2, YY1 siRNA #3, or control siRNA. (G) Difference in the ratio of LC3-II:LC3-I levels induced by transfection with an ADCY3 expression plasmid or the corresponding control vector. (H) Changes in the conversion of LC3-I to LC3-II in C17.2 cells transfected with ADCY3 siRNA #3 or control siRNA. (I) Changes in LC3-I and LC3-II protein levels after transfection with miR-141-3p mimics, with or without the YY1 expression plasmid. (J) Changes in the conversion of LC3-I to LC3-II after transfection with the miR-141-3p inhibitor, with or without YY1 siRNA #2. The data shown indicate the mean values of triplicate experiments ± SD. ∗p < 0.05, ∗∗p < 0.01, relative to the control group.

Apoptosis and autophagy are important self-destructive processes that maintain cellular homeostasis.^18^ To address whether homeostasis was destroyed by imbalanced apoptosis and autophagy, we detected biochemical parameters of autophagy (conversion of microtubule-associated protein 1 light-chain 3 alpha [LC3-I–LC3-II]) and verified that autophagy decreased in the lumbosacral spinal cords of fetal rats with ETU-induced ARMs (Figure 6A). To explore whether miR-141-3p-dependent inhibition of the YY1-ADCY3 axis impaired autophagy, we transfected C17.2 cells with an miR-141-3p mimic. Western blot analysis showed that autophagy decreased (Figure 6B) and transmission electron microscopy (TEM) analysis revealed that fewer autolysosomes were present in C17.2 cells transfected with the miR-141-3p mimic (Figure 6C). Conversely, autophagy increased after transfection with the miR-141-3p inhibitor, as revealed by an increase in the LC3-II:LC3-I ratio (Figure 6D). Transiently overexpressing YY1 in cultured C17.2 cells resulted in increased LC3-II expression (Figure 6E), and LC3-II expression decreased when YY1 expression was silenced, especially with siYY1 #3 (Figure 6F).

Next, we explored the effect of ADCY3 on autophagy induction, which has not been reported previously. As expected, ADCY3 overexpression induced LC3-II expression (Figure 6G), and ADCY3 silencing with siADCY3 #2 and siADCY3 #3 weakened autophagy (Figure 6H). The decreased conversion of LC3-I to LC3-II, induced by miR-141-3p overexpression, was abrogated by YY1 overexpression (Figure 6I). Consistently, miR-141-3p inhibition significantly increased the LC3-II:LC3-I ratio, which was abolished by YY1 knockdown (Figure 6J). Collectively, these results demonstrate that miR-141-3p impaired autophagy by inhibiting the YY1-ADCY3 axis in neural stem cells.

### Homeostasis was restored by decreasing apoptosis and increasing autophagy after intraamniotic anti-miR-141-3p injection through targeting YY1 and ADCY3

**Figure 7:**
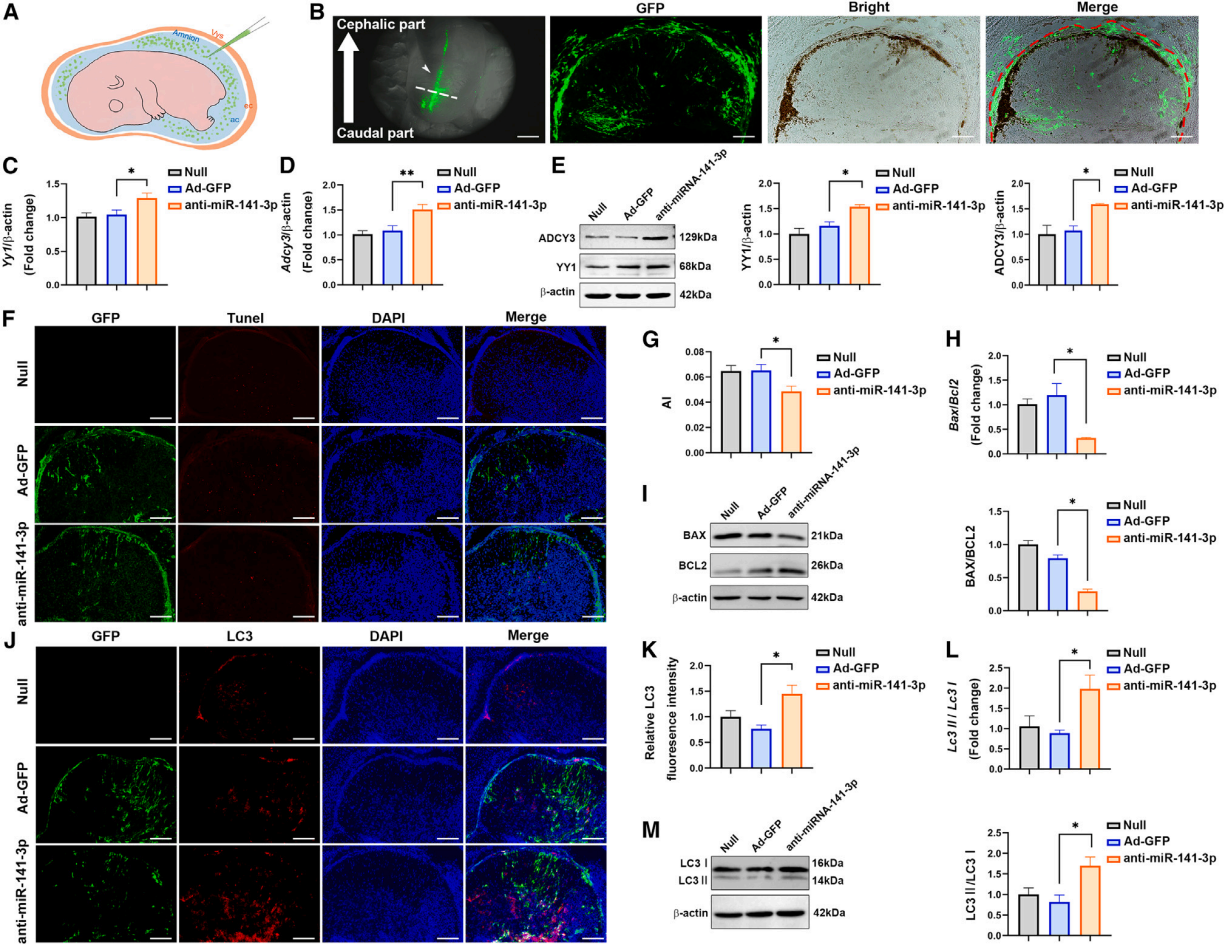
Homeostasis was restored by reducing excessive apoptosis and enhancing impaired autophagy after intraamniotic anti-miR-141-3p injection (A) Schematic representation of the procedure used for intraamniotic anti-miR-141-3p microinjection into E15 embryos. The microinjection pipette (blue) traversed the visceral yolk sac (Vys, orange), exocoelomic cavity (ec), and amnion (green) before entering the amniotic cavity (ac). (B) Left: representative fluorescent stereomicroscopic images of the back of the E21 ARMs fetus in the anti-miR-141-3p group (white arrow indicates the direction of the fetus from caudal part to cephalic part) at 10× (scale bar, 50 μm). Green fluorescence was observed on the back of the fetal neural tube. The white dotted line indicates the location dissected for the transverse section of the spinal cord, which is shown at right. The region indicated by the red curved dotted line is microscopic images of the transverse section of the lumbosacral spinal cord, where GFP reporter (anti-miR-141-3p-sponge) was distributed throughout the whole cross-section, including spinal meninges, spinal cord parenchyma, and the central canal. Scale bar, 50 μm (cryosection images). (C and D) Relative mRNA expression levels of *Yy1* (C) and *Adcy3* (D) in the lumbosacral spinal cords of rat fetuses at E21 in the null (n = 10), Ad-GFP-injected (n = 9), and anti-miR-141-3p-injected group (n = 9) groups. (E) Immunoblot analysis of YY1 and ADCY3 protein expression in the spinal cords of rats in the null (n = 10), Ad-GFP-injected (n = 9), and anti-miR-141-3p-injected group (n = 9) groups at E21. (F) Apoptotic cells (red) are shown by representative confocal microscopic images of the spinal cords of rat fetuses in the ARMs, Ad-GFP-injected, and anti-miR-141-3p-injected groups at E21 using the TUNEL assay at 10× (scale bar, 50 μm). (G) The comparison of AI through the whole transverse section between the null (n = 9), Ad-GFP-injected (n = 9), and anti-miR-141-3p-injected (n = 9) groups. (H) Relative mRNA expression levels of *Bcl2* and *Bax* in the lumbosacral spinal cords of rat fetuses at E21 in the null (n = 10), Ad-GFP-injected (n = 9), and anti-miR-141-3p-injected (n = 9) groups. The values shown represent fold-expression differences in the anti-miR-141-3p-injected groups relative to those in the Ad-GFP-injected and null groups. (I) Immunoblot analysis of BCL2 and BAX protein expression in the spinal cords of rats in the null (n = 10), Ad-GFP-injected (n = 9), and anti-miR-141-3p-injected (n = 9) groups at E21. (J) Fluorescence intensity of LC3 (red) in the spinal cords of rat fetuses in the ARMs, Ad-GFP-injected, and anti-miR-141-3p-injected groups at E21 at 10× (scale bar, 50 μm). (K) Comparison of fluorescence intensity of LC3 puncta through the whole transverse section between the null (n = 9), Ad-GFP-injected (n = 9), and anti-miR-141-3p-injected (n = 9) groups. (L) Relative *Lc3* mRNA in the spinal cords of rat fetuses at E21 in the null (n = 10), Ad-GFP-injected (n = 9), and anti-miR-141-3p-injected (n = 9) groups. The values shown represent fold-expression differences in the anti-miR-141-3p-injected groups when compared with the Ad-GFP and null groups. (M) Immunoblot analysis of the conversion of LC3-I to LC3-II in the spinal cords of rat fetuses in the null (n = 10), Ad-GFP-injected (n = 9), and anti-miR-141-3p-injected (n = 9) groups at E21. All of the experiments were repeated 3 times. ∗p < 0.05, ∗∗p < 0.01, relative to the control group.

To explore whether homeostasis between apoptosis and autophagy may be restored by suppressing miR-141-3p expression during embryogenesis, GFP-labeled anti-miR-141-3p was injected into the amniotic cavity of rat fetuses with ARMs at E15 (Figure 7A). The observation of GFP expression in the fetal spinal cords by fluorescence stereomicroscopy indicated that anti-miR-141-3p transfected effectively (Figure 7B). Furthermore, GFPs in the transverse section of the lumbosacral spinal cords were distributed widely in the spinal membrane, spinal cord parenchyma, and central canal, indicating that anti-miR-141-3p-sponge diffused throughout in the whole spinal cord (Figure 7B). Subsequentially, the function of anti-miR-141-3p-sponge inhibition was investigated. The mRNA and protein expression levels of YY1 and ADCY3 were measured, which presented as significantly higher levels in the anti-miR-141-3p-injected group than those in the null group or a control group injected with an adeno-5 vector that expressed GFP alone (Figures 7C–7E). To clarify whether intraamniotic anti-miR-141-3p injection ameliorated excessive apoptosis and restored impaired autophagy in fetuses with ARMs, cellular apoptosis and autophagy were evaluated in the spinal cord by performing TUNEL and LC3 immunofluorescence staining. We observed significantly less TUNEL^+^ and more LC3^+^ cells in the transverse section of the lumbosacral spinal cords in the anti-miR-141-3p-sponge injection group, especially in the region of GFP^+^ cells, where changes were more pronounced (Figures 7F, 7G, 7J, and 7K). In addition, changes were more pronounced in the region of GFP^+^ cells analyzed by comparison of the apoptosis index (AI) and the fluorescence intensity of LC3 puncta in the GFP expression region and the non-GFP expression region between the Ad-GFP-injected and anti-miR-141-3p-injected groups (Figure S2). Furthermore, the mRNA and protein expression levels of apoptosis and autophagy markers were measured. The mRNA and protein expression levels of BAX and the BAX:BCL2 ratio in the anti-miR-141-3p-injected group were significantly lower than those in the null group or a control group injected with an adeno-5 vector that expressed GFP alone (Ad-GFP group; Figures 7H and 7I). Higher *Lc3* mRNA levels were detected in the anti-miR-141-3p-injected group than in the Ad-GFP and null groups (Figure 7L). A significantly increased LC3-II:LC3-I ratio was observed in anti-miR-141-3p-injected group than the Ad-GFP-injected and ARMs groups (Figure 7M). Collectively, our findings demonstrate that intraamniotic anti-miR-141-3p injection alleviated disordered homeostasis between apoptosis and autophagy during embryogenesis of the lumbosacral spinal cords in fetal rats with ARMs.

## Discussion

Despite the recent progress in elucidating the etiology of postoperative defecation dysfunction in patients with ARMs, the precise mechanisms are far from understood. The control center of defecation is located in the lumbosacral spinal cord, and dysplasia may be a cause of defecation disorders after surgical treatment for ARMs.^6^ Previously, we demonstrated that excessive apoptosis occurred in the lumbosacral spinal cords of fetal rats with ARMs during embryogenesis.^6^ Homeostasis is maintained by a balance between autophagy and apoptosis and plays a crucial role in fetal development.^19^ In this study, we observed that decreased autophagy combined with excessive apoptosis caused imbalanced homeostasis in fetal rats with ARMs. In this study, we observed abnormally elevated miR-141-3p levels in ARMs and confirmed that miR-141-3p played a key role in neurogenesis. Specifically, overexpressing miR-141-3p inhibited YY1 expression by binding to *Yy1* mRNA. Decreased YY1 expression reduced ADCY3 expression via transcriptional regulation, as determined in YY1 ChIP-seq screening experiments, which in turn led to impaired homeostasis due to decreased autophagy and excessive apoptosis. We generated the evidence that miR-141-3p affects homeostasis during spinal cord development by performing intrauterine injections with anti-miR-141-3p in fetal rats with ARMs. These results indicate a key role for intraamniotic anti-miR-141-3p injection and highlight the miR-141-3p-YY1-ADCY3 axis in maintaining the balance between autophagy and apoptosis in ARMs embryos.

miR-141-3p has recently emerged as a key regulator required in the CNS for multiple biological functions,^20^ and miR-141-3p serves dual functions in apoptosis and autophagy.^11^^,^^13^^,^^15^^,^^16^ However, no previous studies have investigated the effects of miR-141-3p on apoptosis and autophagy in rat fetuses with ARMs. The current findings demonstrate a critical role for miR-141-3p in maintaining homeostasis in the central nervous system of defecation and reveal that miR-141-3p overexpression in ARMs triggered apoptosis and repressed autophagy. As a small noncoding RNA, miR-141-3p can bind the mRNA of target genes through their 3′ UTRs and inhibit their expression. Here, as predicted by TargetScan, we discovered that miR-141-3p can bind to the 3ʹ UTR of *Yy1* mRNA at nucleotides 659–665 to block *Yy1* translation.

YY1 is a multifunctional zinc finger-containing transcription factor that plays crucial roles in numerous biological processes by selectively activating or repressing transcription. YY1 is a key gene required for the development of central nervous system.^17^ During the early stages of brain development, YY1 maintains the proliferation and survival of neural progenitor cells by transcriptionally regulating a wide range of metabolic pathways.^21^ Acetylated YY1 binding to both enhancer and promoter regions is essential for OTX2 expression in the anterior neuroectoderm, which plays essential roles at each step and in every tissue during mouse head development, and these YY1-binding sites are highly conserved in enhancers in tetrapods.^22^

Complete ablation of YY1 in mice resulted in early embryonic lethality, and a small subset (16.7%–24%) of YY1 heterozygotes displayed growth retardation and neurulation defects.^17^ The results of another study showed that YY1 mutations led to congenital haploinsufficiency syndrome, which is characterized by craniofacial dysmorphisms and neurological dysfunctions.^23^ Furthermore, YY1 played a pivotal role in embryogenesis by regulating apoptosis. Massive apoptosis was observed in the lung epithelium of *Yy1*^flox/flox^ mouse embryos showing neonatal death due to respiratory failure.^24^^,^^25^ YY1 can also regulate autophagy during development and accelerate oocyte autophagy by transcriptionally activating the long noncoding RNA X-inactive-specific transcript, which binds to pre-miR-23b/pre-miR-29a and prevents its translocation to the cytoplasm.^25^ However, no studies have elucidated the effect of YY1 on homeostasis between apoptosis and autophagy during the development of the central nervous system and the morphogenesis of ARMs. Therefore, we propose a mechanism wherein YY1 downregulation increases apoptosis and represses autophagy, and elevated YY1 protein expression can reverse the imbalance between apoptosis and autophagy induced by high miR-141-3p expression, which contributes to the maldevelopment of the lumbosacral spinal cords of rats with ARMs.

YY1 is a transcription factor that serves dual functions by activating or repressing gene expression. It recognizes and binds to specific DNA sequences through its COOH-terminal domain, which harbors four C2H2-type zinc-finger motifs. Transcriptional regulation of YY1 plays a key role in organogenesis during embryonic development.^26^ Therefore, we explored target genes transcriptionally that are regulated by YY1 by performing ChIP-seq analysis with C17.2 cells. Previous data showed that YY1 recognized a core consensus motif (ATGG) and the structural context of the interferon-β domain in both human and mouse cells.^27^ Luciferase reporter and ChIP assays conducted in mouse neural stem cells further confirmed that YY1 directly interacted with the ATGG motif and the GAGATGG motif in the *Adcy3* promoter and promoted *Adcy3* transcription.

ADCY3 encodes an adenylate cyclase protein, which catalyzes synthesis of the important second messenger cyclic AMP and has been previously implicated in obesity.^28^ Homozygous *Adcy3* mutations were identified in affected children in a cohort of severely obese consanguineous individuals from Pakistan.^29^ ADCY3 localized to primary cilia throughout the brain, and *Adcy3*^flox/flox^ mice became obese after administration of an AAV-CRE-GFP vector into the hypothalamus.^30^^,^^31^ ADCY3 expression was downregulated in *Olig2*-knockout mice and *Olig1/Olig2* double-knockout mice, which showed compromised terminal differentiation and maturation of olfactory sensory neurons (OSNs), and excessive OSNs apoptosis has been reported in *Adcy3*^−/−^ mice.^32^^,^^33^ In addition, ADCY3 decreased apoptosis in gastric cancer cells by functioning as competing endogenous RNAs.^34^ However, ADCY3 has not been associated with autophagy during embryogenesis. In this study, we demonstrated a function for ADCY3 in ARMs during rat embryogenesis. ADCY3 was downregulated in the lumbosacral spinal cords of rat fetuses with ARMs and was transcriptionally regulated by YY1. ADCY3 was also important for maintaining homeostasis between apoptosis and autophagy. These results suggested that ADCY3 expression, which is regulated by miR-141-3p and YY1, is probably involved in the dysfunction of the defecation control center in rat embryos with ARMs.

Few studies have focused on injecting cells and molecules into the amniotic cavity to treat congenital anomalies during embryogenesis. Human mesenchymal stem cells (hMSCs) were used to treat rats with nitrogen-induced congenital diaphragmatic hernias (CDH-hMSCs group), which showed a higher survival rate, larger air spaces, and thinner alveolar walls than those in a control CDH group.^35^ Previously, we demonstrated that intraamniotic bone marrow-derived mesenchymal stem cell (BMSC) injections into rats during the early development of neural tube defects (NTDs) may promote functional recovery.^36^ In further studies, we treated rats with NTDs with intraamniotic microinjection of BMSCs and collapsin response mediator protein 4 (CRMP4) siRNA. The therapeutic effects in the combined-treatment group were superior to those of the CRMP4 siRNA or BMSC group in terms of reduced skin lesion areas and higher amplitude of motor-evoked potentials.^37^ In addition, we found that injecting an miR-322 mimic into the amniotic cavity attenuated apoptosis by silencing NOX4 in a mouse model of all-*trans* retinoic acid-induced NTDs.^38^ In a previous study, vectors encoding either an mmu (*Mus musculus*)-miR-141 inhibitor or an mmu-miR-141 mimic injected into mouse uteri decreased the number of embryo implantation sites.^39^ In another study, nanoparticles with encapsulated peptide nucleic acid-based or phosphorothioate-based anti-miR-141-3p probes showed potential in mice as treatments for ischemic stroke.^40^ However, intraamniotic injection of miRNAs has not yet been reported for the treatment of ARMs. Therefore, in this study, intraamniotic injection of anti-miR-141-3p was performed in a fetal rat model of ETU-induced ARMs. The anti-miR-141-3p was microRNA sponge bearing specific binding sites in its tandem repeats of identical sequence on the 3ʹ UTR of miR-141-3p delivered by adenoviral GFP vector.^41^ Delivery of sponge constructs with viral vectors to tissue is feasible and is convenient to make dominant-negative transgenic expression.^41^^,^^42^ Our results showed that anti-miR-141-3p injected into the amniotic cavity diffused into the neural tube successfully using adenoviral GFP vector. Notably, the unintended “off-target” silencing of endogenous genes created by GFP reporter should be ruled out by comparing phenotypes between the no-treatment, control vector treatment, and miRNA sponge groups, respectively.^43^ The present study has revealed no significant expression changes of apoptosis (BAX:BCL2 ratio and TUNEL) and autophagy (LC3-II:LC3-I ratio) between the no-treatment group and control vector treatment group, whereas the data from both groups were significantly different from the anti-miR-141-3p injected group. These data exhibited the restoration efficiency of anti-miR-141-3p in maintaining homeostasis in the defecation center of ARM fetal rats and no significant off-target effect of GFP reporter *in vivo* injection using anti-miR-141-3p delivered by adenoviral GFP vector primarily, indicating that anti-miR-141-3p creates a more permissive environment for neuronal survival in the defective spinal cords of fetal rats with ARMs. However, more experiments should be conducted to evaluate the safety and off-target effects of sponges delivered by adenoviral GFP vector before it can be translated to clinical practice.

Our results highlight the *in vivo* role of miR-141-3p during organogenesis of the lumbosacral spinal cord and the role of the miR-141-3p-YY1-ADCY3 axis in managing autophagy and apoptosis in ARMs . The efficacy of anti-miR-141-3p is mainly related to its ability to decrease apoptosis and increase autophagy, which helped maintain homeostasis between apoptosis and autophagy in the lumbosacral spinal cords of rats with ARMs during embryogenesis. As the studies related above,^36^^,^^37^ BMSCs were demonstrated to transdifferentiate into many kinds of neural cells for treatment, it would be more conducive to further promoting the differentiation and survival of neurons in the lumbosacral spinal cords of ARMs fetuses with intraamniotic injection of BMSCs combined with anti-miR-141-3p in future studies. In addition, due to that the defecation function of ARMs is affected by the lumbosacral spinal cord defecation center, the pelvic floor muscles, and the hindgut, the expression and function of miR-141-3p were further investigated in the other two kinds of tissues in the future. Collectively, these results demonstrate the therapeutic potential of intraamniotic injection of anti-miR-141-3p for treating postoperative defecation dysfunction in ARMs. miR-141-3p regulates autophagy and apoptosis through the miR-141-3p-YY1-ADCY3 axis, which provides a mechanistic basis for embryonic neural development in the lumbosacral spinal cords of rat fetuses with ARMs. These promising findings should be explored in combination with stem cell therapy.

## Materials and methods

### Animal model and tissue collection

Outbred Wistar rats aged 10–12 weeks were obtained from the Medical Animal Center of Shengjing Hospital (China Medical University, Shenyang, Liaoning, China). This study was approved by the Animal Ethics Committee of the Shengjing Hospital of China Medical University (approval no. 2022PS360K). The rat model of ARMs was established using ETU. After mating with a male rat, female rats’ vaginal plugs and smears were examined. If sperm cells were present in a vaginal smear, then the day was recorded as E0. Pregnant rats (body mass, 250–300 g) were divided into two groups, in which one group received a single intragastric administration of 1% (w/v) ETU (Sigma-Aldrich, Penzberg, Germany) in saline (125 mg/kg body weight) on E10 via gavage feeding. Pregnant rats in the control group received an equal volume of saline. Embryos were harvested by cesarean delivery on E17, and the normal fetal rats harvested in the control group on E17 was indicated as N17. The ARMs group (ETU-fed rats with ARMs) on E17 included in this experiment was named A17, and those with other defects, such as encephaloceles, meningoceles, and occult spina bifida, were excluded from the analysis. Lumbosacral spinal cords were dissected under a stereomicroscope, immediately frozen in liquid nitrogen, and stored at −80°C in preparation for qRT-PCR and western blot analyses.

### Reagents and plasmid construction

miR-141-3p mimic, miR-141-3p inhibitor, and appropriate negative control were purchased from RIBOBIP (Guangzhou, China). We amplified and subcloned the 3′ UTR of *Yy1* and a portion of the predicted miR-141-3p-binding site in its 3′ UTR and inserted these sequences into the pmirGLO Dual-Luciferase miRNA Target Expression Vector (Promega, Madison, WI), thereby generating the 3′ UTR-Luc and binding site-Luc constructs (Liaoning Bai Hao Biological Technology, Benxi, Liaoning, China.). Then, the *Yy1* 3′ UTR binding site was subsequently removed by introducing an internal mutation to generate the Mut-Luc construct (Liaoning Bai Hao Biological Technology). Primers for amplifying miR-141-3p, U6, *Yy1*, and *β-actin* was made by Sangong Biotech (Shanghai, China). siRNAs against *Yy1* and *Adcy3* mRNAs, expression plasmids encoding YY1 or ADCY3, and negative controls were purchased from HanBio Therapeutics (Shanghai, China).

### Cell culture and transfections

C17.2 mouse neural stem cells were obtained from the Beina Chuanglian Biology Research Institute and cultured in minimum essential medium (MEM; Gibco, Waltham, MA) supplemented with 1% nonessential amino acids (Gibco), 10% fetal bovine serum (FBS, Gibco), 100 U/mL (1%) penicillin, and 100 μg/mL (1%) streptomycin in a humidified incubator at 37°C with 5% CO_2_. C17.2 cells were seeded into 6-well plates at a density of 1 × 10^5^ cells/well and cultured for 24 h to a purity of >90% for transfection. Lipofectamine 3000 (5 μL) (Thermo Fisher Scientific, Waltham, MA) mixed with 100 pmol miR-141 mimic, miR-141 inhibitor, or negative controls (Guangzhou RiboBio, Guangzhou, China) were added into 125 μL Opti-MEM (Invitrogen, Carlsbad, CA) separately and incubated for 10 min at room temperature under serum-free conditions to form transfection complexes, according to the manufacturer’s instructions. The C17.2 cells were plated in 6-well plates at a density of 5,000 cells/well after being washed twice with PBS and then added to the corresponding transfection complexes. Complete MEM media was replaced 6 h later. The transfection efficiency was checked under a fluorescence microscope. After the treatments, the cells were used in the subsequent experiments.

### TEM analysis

Cells collected after transfection of miR-141-3p mimic and the control mimic for 48 h were centrifuged at 1,200 rpm/min for 5 min and fixed with 4% glutaraldehyde overnight at 4°C. The cells were then washed 3 times with 0.1 M PBS (15 min/wash) and refixed in 1% osmic acid for 1 h at 4°C, followed by 3 washes with double-distilled H_2_O (15 min each). Then, the cell samples were embedded for 4 h at 20°C, incubated overnight at 37°C, and cured in an oven at 60°C for 24 h. The samples were then sectioned and stained with 1% uranyl acetate for 20 min, followed by staining with lead citrate for 5 min. Ultrathin sections (70 nm thick) were prepared and *in vitro* images were acquired using a JEM-1400Plus TEM (JEOL, Tokyo, Japan) operated at 80 kV. During TEM analysis, we distinguished autophagosomes as vesicles with double-membrane structures engulfing cytoplasmic material.

### Dual-luciferase reporter assay

PcDNA3.1 vectors, containing different *Adcy3* promoter sequences, were cotransfected into C17.2 cells with or without the YY1 plasmid. The indicated YY1 luciferase reporter vector was cotransfected into the C17.2 cells with the miR-141-3p mimic or the control mimic. The cells were harvested after 48 h, and cell lysates were collected for firefly and Renilla luciferase assays using the Dual-Luciferase Reporter Assay System (Promega) according to the manufacturer’s protocol and normalized to Renilla luciferase activity.

### ChIP-seq and ChIP-qPCR analyses

For ChIP-seq analysis of YY1, a DNA library was prepared and sequenced (300 cycles) on an Illumina NovaSeq 6000 instrument using the NovaSeq 6000 S4 Reagent Kit (Illumina, San Diego, CA), according to the manufacturer’s protocol. Illumina Pipeline software was used for sequencing analysis, and the sequences were aligned to the mouse genome (UCSC mm10). Aligned reads were used for peak calling with ChIP sequences using MACS software (version 1.4.2). Statistically significant ChIP-enriched regions (peaks) were identified by comparing the ChIP-seq data with input sequence data using a p value threshold of 10^−3^. All 10-bp-resolution ChIP-seq profiles for each sample were saved in UCSC Wig format for visualization with the UCSC Genome Browser.

ChIP-qPCR was performed using the SimpleChIP Enzymatic Chromatin IP Kit (Cell Signaling Technology, catalog no. 9005, Danvers, MA) according to the manufacturer’s recommended protocol. Briefly, C17.2 cells were crosslinked with formaldehyde and lysed with cell lysis buffer, followed by sonication to an average size of 150–900 bp. Thereafter, the chromatin extracts were immunoprecipitated overnight using an anti-YY1 antibody (Cell Signaling Technology, catalog no. 11911) or a negative-control antibody normal rabbit immunoglobulin G (IgG; Cell Signaling Technology, catalog no. 2729). The recovered DNA was subjected to qRT-PCR analysis to amplify the YY1 binding site in the *Adcy3* promoter fragment. The following primers were used to amplify DNA containing motif 1 (GGAGAGATGGCT): forward, 5′-GCACGTTTTCTTTTCAGCTTGGA-3′, and reverse, 5′- TCTTTCCTTGACCCAACCTACC-3′. The following primers were used to amplify DNA containing motif 2 (TGTAGCATGGAT): forward, 5′- ATTTAGGTGAGGAAGTCAGGGA-3′, and reverse, 5′-ACCGAACATCTGCAACTTAAAAGG-3′.

### Intraamniotic microinjections

Fetal rats with ETU-induced ARMs and control rat fetuses were administered intraamniotic injections on E15, as described in one of our previous studies.^44^ Under pentobarbital sodium anesthesia (40 mg/kg body weight), an incision was made in the abdominal wall of pregnant rats, the uterus was exposed, and the fetal rat morphology was observed under an operating microscope (Möller, Hamburg, Germany) through the translucent uterine wall. Fetuses with ARMs were randomly divided into PBS-injected (null), Ad-GFP injected (5.01 × 10^11^ plaque-forming units [PFUs]/mL; HanBio Therapeutics), and Ad-rno-miR-141-3p-sponge-GFP-injected (anti-miR-141-3p injected; 5.01 × 10^11^ PFUs/mL; HanBio Therapeutics) groups. Microinjections were performed via intraamniotic injection of 5 μL of solution using a glass micropipette (internal tip diameter, 100 mm) connected to a Hamilton syringe. The micropipettes used for the injections were made from borosilicate glass capillaries (model GD-1; Narishige Scientific Instruments, Tokyo, Japan) using a micropipette puller (model PB-7; Narishige Scientific Instruments). After the injections, each uterus was returned to the abdomen, and the abdominal incision was closed in two layers. Pregnant rats recovered from anesthesia within 1 h and were returned to their home cages. To promote survival of the fetuses, we injected only two or three fetuses per dam. Pregnant rats were euthanized at E21 using an overdose of pentobarbital sodium, and the treated fetuses were harvested for analysis. The lumbosacral spinal cord was dissected under a stereomicroscope, immediately frozen in liquid nitrogen, and stored at −80°C in preparation for qRT-PCR and western blot analysis. Vertebrae with spinal cords were harvested and fixed overnight at 4°C in 0.1 M PBS containing 4% paraformaldehyde, after which they were cryoprotected in 20% sucrose for 24 h, embedded in optimal cutting temperature compound, and sectioned into 25-μm serial sections using a freezing microtome (Microm hm525, Thermo Fisher Scientific). Finally, the sections were subjected to immunohistochemical staining.

### FISH

FISH of miR-141-3p was performed in 5.0-μm formalin-fixed paraffin-embedded tissue sections with a 5′-CY3-labeled probe (Servicebio, Wuhan, China). The sequence of the miR-141-3p probe is listed in Table S3. Briefly, after deparaffinization in xylene and rehydration in graded ethanol solutions (100%, 85%, and 75%), tissue sections were antigen repaired by boiling in 100°C of the repair solution and continued for 15 min. Then, the sections were digested with Proteinase K (Servicebio) for 30 min and prehybridized for 30 min at 40°C. Subsequently, after being hybridized overnight at 40°C, the sections were counterstained with DAPI (Servicebio) and incubated in the dark for 8 min. Finally, the sections were sealed using antifluorescence quenching sealing tablets (Servicebio). A confocal laser microscope (LSM 880, Zeiss, Oberkochen, Germany) was used to capture red light emission at 550–570 nm. Average optical density (AOD) analysis was performed using Image-Pro Plus software (version 6.0; Media Cybernetics, Rockville, MD).

### Immunofluorescence staining

Sections of fetal spinal cords obtained on E21 were permeabilized with Proteinase K and blocked with PBS containing 10% FBS and 0.1% Triton X-100. The primary antibody used for immunofluorescence analysis was directed against LC3A/B (1:200 dilution; Cell Signaling Technology, catalog no. 4108). The sections were stained with secondary Alexa Fluor 555-conjugated goat anti-rabbit or anti-mouse IgG antibodies (1:100; Invitrogen) for 1.5 h at 37°C. After washing the sections, they were stained with DAPI and mounted using Anti-Fade Mounting Medium. Images were captured using a C1 confocal microscope (Nikon, Tokyo, Japan).

Moreover, TUNEL staining was also applied to evaluate the apoptosis of tissue by a TUNEL assay kit (Beyotime, catalog no. C1090) following the manufacturer’s instructions. Spinal cord staining was fixed with 4% paraformaldehyde, blocked with 0.5% Triton X-100 PBS, and staining was performed at 37°C for 1 h. DAPI staining was performed for the recognition of nuclei and the categorization of cells. Apoptotic cells and LC3^+^ cells were visualized and imaged using a fluorescence microscope (Nikon ECLIPSE 80i). The number of total TUNEL^+^ cells in the whole cross-section was counted under 40× magnification, and then AI was determined and reported as the number of TUNEL^+^ cells per total cells in each group. The images captured for LC3^+^ cells under 40× magnification were analyzed for fluorescence intensity of LC3 puncta in the whole cross-section using ImageJ software. Subsequently, we demarcated the fluorescent and nonfluorescent regions, and analyzed the AI and fluorescence intensity of LC3 puncta in them separately.

### Combined FISH/immunostaining

Combined FISH/immunostaining was performed as described previously.^45^ Briefly, after deparaffinization in xylene and rehydration in graded ethanol solutions (100%, 85%, and 75%), the sections were digested with Proteinase K (Servicebio) for 20 min and prehybridized for 30 min at 40°C. Then, the sections were hybridized overnight at 40°C. After abandoning the hybrid solution, it was washed by gradient saline sodium citrate buffer at 37°C for 5 min successively. Spinal sections were washed several times with PBS, blocked by incubation in goat serum at 37°C for 30 min, and then immunolabeled by incubation with NESTIN antibody (1:200; catalog no. MAB353, EMD Millipore, Darmstadt, Germany) overnight at 4°C. Samples were then incubated with corresponding secondary antibodies for 1 h, treated with antifluorescence quenching tablets containing DAPI for counterstaining, and imaged under a confocal fluorescence microscope.

### Western blotting

Proteins were extracted from rat fetal spinal cord tissues and mouse neural stem cells (C17.2). Approximately 40 μg protein from each sample type was separated by SDS-PAGE, followed by electrotransfer to a polyvinylidene difluoride membrane. A nonfat milk solution (5%) was used for blocking before an overnight incubation at 4°C with primary antibodies against YY1 (1:1,000; catalog no. DF8072, Affinity Biosciences, Jiangsu, China), ADCY3 (1:1,000; catalog no. 19492-1-AP, Proteintech, Wuhan, China), LC3 (1:1,000; catalog no. 4108, Cell Signaling Technology), BAX (1:1,000; catalog no. 2772, Cell Signaling Technology), or BCL-2 (1:1,000; catalog no. B9804, Sigma-Aldrich, St. Louis, MO). Membranes were washed 3 times (10 min/wash) with Tris-buffered saline plus Tween-20) and then incubated with a secondary antibody (1:5,000; Invitrogen) at room temperature for 2 h. Enhanced chemiluminescence reagent (Millipore, Burlington, MA) was used to detect protein-antibody interactions. Protein bands were analyzed using ImageJ software and normalized to β-actin or glyceraldehyde 3-phosphate dehydrogenase levels.

### miRNA and RNA extraction and qRT-PCR analysis

Total mRNA was extracted from embryos and C17.2 cells using TRIZOL reagent (Takara, Ohtsu, Japan), and miRNAs were extracted using the miRNeasy Mini Kit (Qiagen, Hilden, Germany) according to the manufacturer’s instructions. cDNA was synthesized using the PrimeScript RT Reagent Kit (Takara) and the miRNA First Strand cDNA Synthesis Kit (Takara) with RNA as the template. mRNA and miRNA expression levels were measured using the SYBR Premix Ex Taq Kit (Takara) on a 7500 Real-Time PCR System (StepOnePlus, ABI, Oyster Bay, NY). Relative gene expression levels were calculated using the 2^−▵▵Ct^ method. Primers for amplifying β-actin, U6, and related genes are shown in Table S4.

### Statistical analyses

All of the experiments were performed independently with at least three biological replicates. Statistical analyses and graphing were performed using GraphPad Prism software (version 9.0; GraphPad Software, La Jolla, CA). All of the results are expressed as the mean ± SEM. The Student’s t test (two-tailed) was used to compare two groups with normally distributed data; otherwise, the Mann-Whitney *U* test was used. Pearson’s correlation coefficient was calculated using SPSS (version 26.0; IBM SPSS Statistics, Armonk, NY). p < 0.05 was considered statistically significant.

## Data and code availability

All of the data generated or analyzed during this study are available from the corresponding authors upon reasonable request.

## Supplemental information

Document S1. Tables S1‒S4 and Figures S1 and S2

Document S2. Article plus supplemental information
