# Supplementary material for: Anti-miR-141-3p maintains homeostasis between autophagy and apoptosis by targeting Yy1 in the fetal lumbosacral defecation center of rats
Source: Mol Ther Nucleic Acids. 2024 Mar 6;35(2):102163. doi: 10.1016/j.omtn.2024.102163 (PMC10965809; doi:10.1016/j.omtn.2024.102163)
Supplement: Document S1. Tables S1‒S4 and Figures S1 and S2 [file mmc1.pdf]

**OMTN, Volume *35***

## Supplemental information

# Anti-miR-141-3p maintains homeostasis between autophagy and apoptosis by targeting Yy1 in the fetal lumbosacral defecation center of rats

**Yue Li, Peiqi Liu, Yifan Yao, Weilin Wang, Huimin Jia, Yuzuo Bai, Zhengwei Yuan, and Zhonghua Yang**

**Table S1**

**Supplemental Information for Fig.3C: The GO terms enriched by genes of YY1 binding promoter regions**

| Ontology | ID | Description |
|---|---|---|
| MF | GO:0005544 | Calcium-dependent phospholipid binding |
| MF | GO:0016755 | Transferase activity, transferring amino-acyl groups |
| MF | GO:0098772 | Molecular function regulator |
| MF | GO:0019899 | Enzyme binding |
| MF | GO:0042562 | Hormone binding |
| MF | GO:0019900 | Kinase binding |
| MF | GO:0019901 | Protein kinase binding |
| MF | GO:0005251 | Delayed rectifier potassium channel activity |
| MF | GO:0005488 | Binding |
| MF | GO:0005132 | Type l interferon receptor binding |
| CC | GO:0043229 | Intracellular organelle |
| CC | GO:0030894 | Replisome |
| CC | GO:0043601 | Nuclear replisome |
| CC | GO:0099503 | Secretory vesicle |
| CC | GO:0030133 | Transport vesicle |
| CC | GO:0070382 | Exocytic vesicle |
| CC | GO:0030688 | Preribosome, small subunit precursor |
| CC | GO:0005662 | DNA replication factor A complex |
| CC | GO:0045095 | Keratin filament |
| CC | GO:0110165 | Cellular anatomical entity |
| BP | GO:0060538 | Skeletal muscle organ development |
| BP | GO:1901862 | Negative regulation of muscle tissue development |
| BP | GO:0007519 | Skeletal muscle tissue development |
| BP | GO:0048635 | Negative regulation of muscle organ development |
| BP | GO:0045843 | Negative regulation of striated muscle tissue |

| | | |
|---|---|---|
| | | development |
| BP | GO:0048641 | Regulation of skeletal muscle tissue development |
| BP | GO:0035914 | Skeletal muscle cell differentiation |
| BP | GO:2001014 | Regulation of skeletal muscle cell differentiation |
| BP | GO:0048642 | Negative regulation of skeletal muscle tissue development |
| BP | GO:2001015 | Negative regulation of skeletal muscle cell differentiation |

MF: Molecular function

CC: Cellular component

BP: Biological process

**Table S2**

**Supplemental Information for Fig.3D: The top 10 pathway enriched terms associated with genes of YY1 binding promoter regions in C17.2 cells**

| Ontology | ID | Description |
|---|---|---|
| KEGG | mmu03008 | Ribosome biogenesis in eukaryotes |
| KEGG | mmu04022 | cGMP-PKG signaling pathway |
| KEGG | mmu03010 | Ribosome |
| KEGG | mmu04926 | Relaxin signaling pathway |
| KEGG | mmu04261 | Adrenergic signaling in cardiomyocytes |
| KEGG | mmu04725 | Cholinergic synapse |
| KEGG | mmu05016 | Huntington disease |
| KEGG | mmu05210 | Colorectal cancer |
| KEGG | mmu04371 | Apelin signaling pathway |
| KEGG | mmu04211 | Longevity regulating pathway |

**Table S3 The sequence of the miR-141-3p probe**

| Term | Sequence (5’-3’) |
|---|---|
| miR-141-3p probe | 5′-CY3-GCCATCTTTACCAGACAGTGTTAGG-3′ |

**Table S4 The sequences of primers used in qRT-PCR**

| mRNA | Accession number | Primer equence (5'-3') | Annealing temperature(℃) |
|---|---|---|---|
| β-actin | NM_031144.3 | Sense: TCAGGTCATCACTATCGGCAAT<br>Antisense: AAAGAAAGGGTGTAAAACGCA | 60 |
| | NM_007393.5 | Sense: GGAGATTACTGCCCTGGCTCCTA<br>Antisense: GACTCATCGTACTCCTGCTTGCTG | 60 |
| Yy1 | NM_173290.2 | Sense: GTGGCAAAGCGTTCGTTGAG<br>Antisense: CGTGCGCAAATTGAAGTCCA | 60 |
| | NM_009537.4 | Sense: GGACGACGACGACGAGGAC<br>Antisense: TGGTGGTGGTGGTGGTGATG | 60 |
| Adcy3 | NM_130779.2 | Sense: GTCGCTGGAGGTGAAGATGAATCTG<br>Antisense: ATGGTGTTGAACTGCTGCTGGTC | 60 |
| | NM_001159536.1 | Sense: GCATGTTGCACGCCATTTTC<br>Antisense: AGTCAGCAAAGTTGGGCAAG | 60 |
| U6 | NC_051340.1 | Sense: TGGAACGCTTCACGAATTTGCG<br>Antisense: GGAACGATACAGAGAAGATTAGC | 60 |
| | NC_000083.7 | Sense: CTCGCTTCGGCAGCACA<br>Antisense: AACGCTTCACGAATTTGCGT | 60 |
| miR-141-3p | NC_051339.1 | Sense: GGTAGAAATGGTCTGTCACAAT | 60 |
| Bcl2 | NM_016993 | Sense: CTACTCGCCTCCTGTCACATCGGGCATCG<br>Antisense: GAGCCTGCCGAGGTGCCGTAGTACA | 60 |
| Bax | NM_017059.2 | Sense: TGGAAGAAGATGGGCTGAGGC<br>Antisense: CATTCCCACCCCTCCCAATAAT | 60 |
| LC3 | NM_012823.2 | Sense: TTCACCGAGATCCTGTGTCTACG<br>Antisense: AATGCTGTCCTCAATGTCCTTCTG | 60 |

| | | |
|---|---|---|
| chip-s1 | Sense: GCACGTTTTCTTTTCAGCTTGGA<br>Antisense: TCTTTCCTTGACCCAACCTACC | 60 |
| chip-s2 | Sense: ATTTAGGTGAGGAAGTCAGGGA<br>Antisense: ACCGAACATCTGCAACTTAAAAGG | 60 |

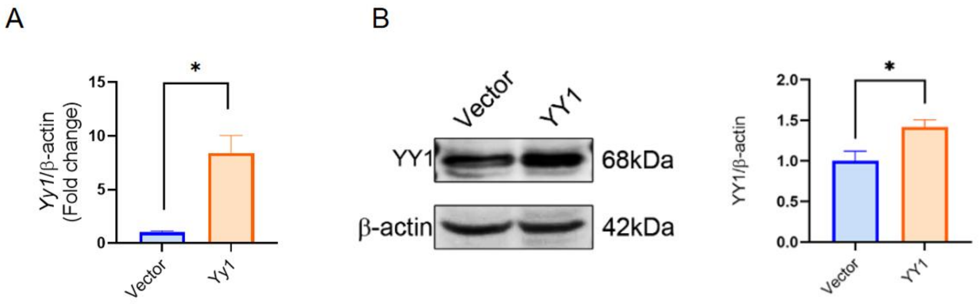


**Figure S1**: (A, B) Changes in the mRNA-expression (A) and protein-expression (B) levels of YY1 in C17.2 neural stem cells after transfecting them with a YY1-expression plasmid or the control vector.

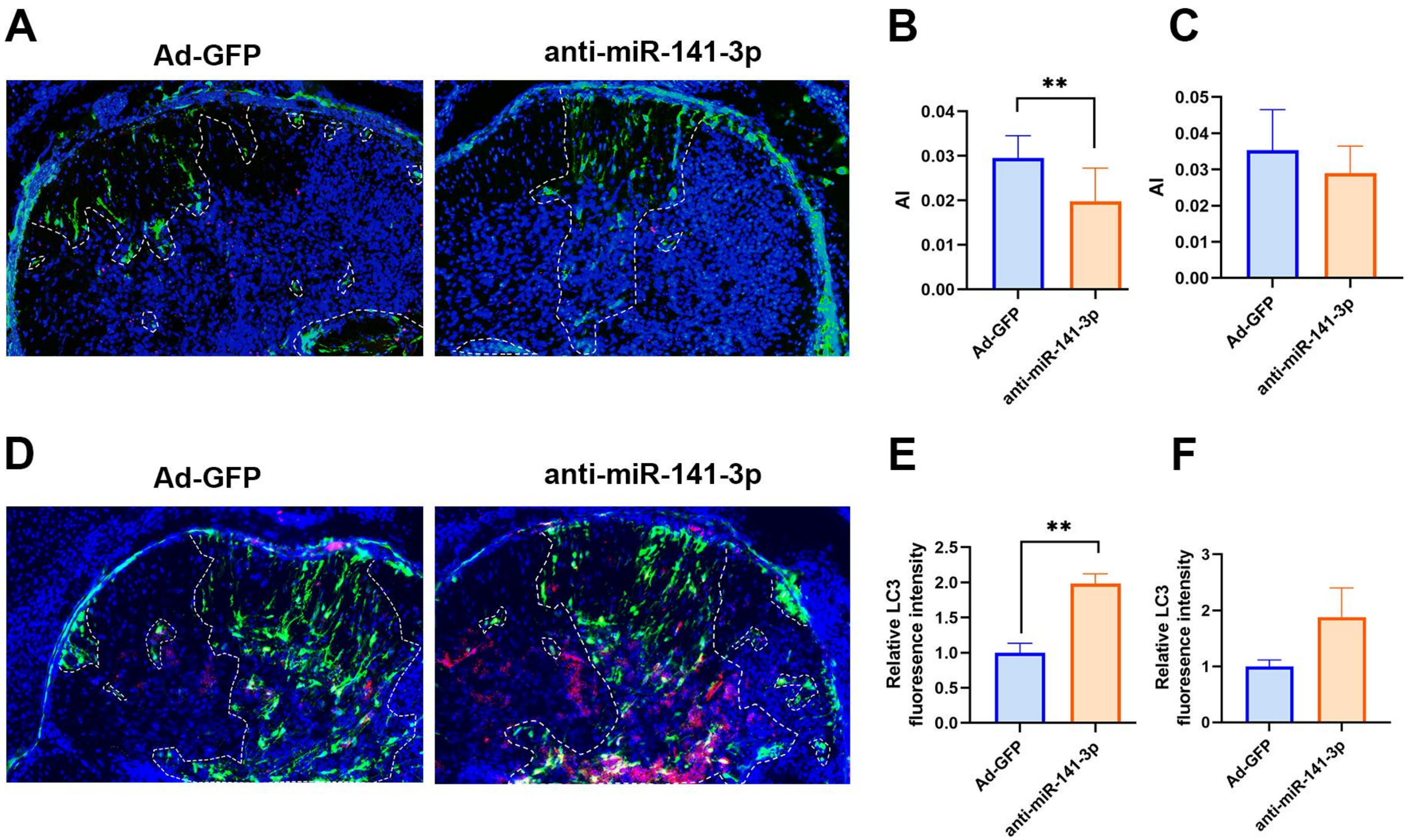
A
Ad-GFP
anti-miR-141-3p
B
**
AI
0.04
0.03
0.02
0.01
0.00
Ad-GFP
anti-miR-141-3p
C
AI
0.05
0.04
0.03
0.02
0.01
0.00
Ad-GFP
anti-miR-141-3p
D
Ad-GFP
anti-miR-141-3p
E
**
Relative LC3
fluoresence intensity
2.5
2.0
1.5
1.0
0.5
0.0
Ad-GFP
anti-miR-141-3p
F
Relative LC3
fluoresence intensity
3
2
1
0
Ad-GFP
anti-miR-141-3p

Figure S2:(A) Apoptotic cells (red) are shown by representative confocal microscopic images of the spinal cord of rat fetuses in the Ad-GFP-injected and anti-miR-141-3p-injected groups at E21 using the TUNEL assay at 10× (Scale bar=50μm).The area in the white dotted line was the GFP expression region, otherwise non-GFP expression regions. (B) The comparison of apoptosis index (AI) in the GFP expression region between Ad-GFP-injected (n =9) and anti-miR-141-3p-injected (n =9) groups.(C) The comparison of apoptosis index (AI) in the non GFP expression region between Ad-GFP-injected (n =9) and anti-miR-141-3p-injected (n =9) groups. (D) Fluorescence intensity of LC3 (red) in the spinal cord of rat fetuses in the , Ad-GFP-injected and anti-miR-141-3p-injected groups at E21 at 10× (Scale bar=50μm).The area in the white dotted line was the GFP expression region, otherwise non-GFP expression regions. (E) Comparison of fluorescence intensity of LC3 punctain the GFP expression region between Ad-GFP-injected (n = 9) and anti-miR-141-3p-injected (n = 9) groups. (F) Relative LC3 mRNA in the spinal cord of rat fetuses at E21 in the Ad-GFP-injected (n = 9) and anti-miR-141-3p-injected (n = 9) groups. All experiments were repeated thrice. *P < 0.05 vs. control group.
